# Efficient Syntheses of Biobased Terephthalic Acid, *p*-Toluic Acid, and *p*-Methylacetophenone via One-Pot Catalytic Aerobic Oxidation of Monoterpene Derived Bio-*p*-cymene

**DOI:** 10.1021/acssuschemeng.1c02605

**Published:** 2021-06-17

**Authors:** Joshua D. Tibbetts, Danilo Russo, Alexei A. Lapkin, Steven D. Bull

**Affiliations:** † Department of Chemistry, University of Bath, Claverton Down, Bath, BA2 7AY, U.K.; ‡ Centre for Sustainable Chemical Technologies, University of Bath, Claverton Down, Bath, BA2 7AY, U.K.; § Department of Chemical Engineering and Biotechnology, University of Cambridge, West Cambridge Site, Philippa Fawcett Drive, Cambridge, CB3 0AS, United Kingdom

**Keywords:** Terpene biorefinery, Bio-*p*-cymene, Bio-*p*-methylacetophenone, Bio-*p*-toluic acid, Bio-terephthalic acid, Catalytic aerobic oxidation

## Abstract

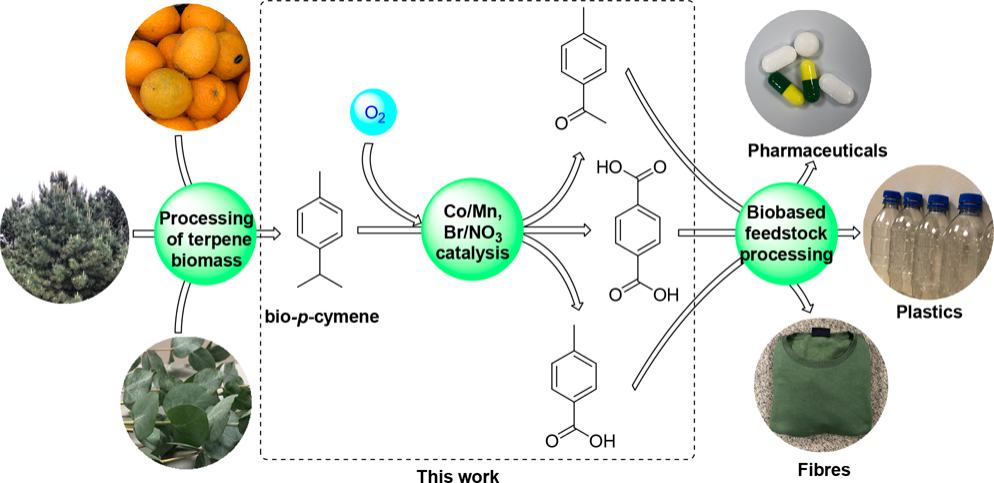

An efficient elevated-pressure catalytic oxidative process (2.5 mol % Co(NO_3_)_2_, 2.5 mol % MnBr_2_, air (30 bar), 125 °C, acetic acid, 6 h) has been developed to oxidize *p*-cymene into crystalline white terephthalic acid (TA) in ∼70% yield. Use of this mixed Co^2+^/Mn^2+^ catalytic system is key to obtaining high 70% yields of TA at relatively low reaction temperatures (125 °C) in short reaction times (6 h), which is likely to be due to the synergistic action of bromine and nitrate radicals in the oxidative process. Recycling studies have demonstrated that the mixed metal catalysts present in recovered mother liquors could be recycled three times in successive *p*-cymene oxidation reactions with no loss in catalytic activity or TA yield. Partial oxidation of *p*-cymene to give *p*-methylacetophenone (*p*-MA) in 55–60% yield can be achieved using a mixed CoBr_2_/Mn(OAc)_2_ catalytic system under 1 atm air for 24 h, while use of Co(NO_3_)_2_/MnBr_2_ under 1 atm O_2_ for 24 h gave *p*-toluic acid in 55–60% yield. Therefore, access to these simple catalytic aerobic conditions enables multiple biorenewable bulk terpene feedstocks (e.g., crude sulfate turpentine, turpentine, cineole, and limonene) to be converted into synthetically useful bio-*p*-MA, bio-*p*-toluic acid, and bio-TA (and hence bio-polyethylene terephthalate) as part of a terpene based biorefinery.

## Abstract 2

Efficient catalytic aerobic oxidative protocols are used to transform terpene-derived bio-*p*-cymene into biorenewable terephthalic acid, *p*-toluic acid, and *p*-methylacetophenone.

## Introduction

**Figure 1:**
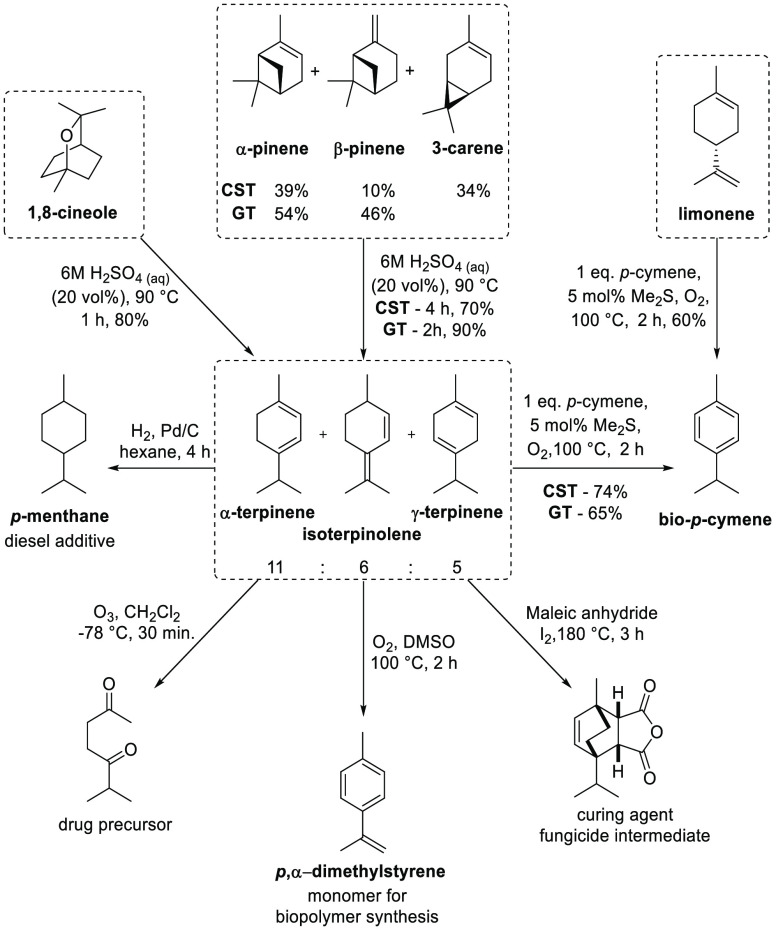
Terpene biorefinery model showing conversion of CST, GT, limonene, and 1,8-cineole into *p*-MeD mixtures that are then valorized into biorenewable chemical products.^8^

Biorefineries are predicted to play an increasingly important role in the sustainable transformation of biomass into the diverse range of chemical products and fuels that are currently sourced from petrochemicals.^1−3^ The forestry industry has pioneered use of the biorefinery concept for more than 150 years, with the Kraft paper making process used to transform wood chips into paper generating significant amounts of other sustainable product streams, including Kraft lignin, crude tall oil, and crude sulfate turpentine (CST).^2^ CST is a useful biorenewable monoterpene feedstock that is comprised of a sulfurous mixture of the bicyclic monoterpenes α-pinene, β-pinene, and 3-carene, as well as smaller amounts of camphene and other monocyclic terpenes (e.g., limonene and terpinolene). CST production from the paper pulping industry accounts for around two-thirds of global turpentine supplies (∼260 000 t year^–1^), with the remainder produced as gum turpentine (GT) through distillation of oleoresin harvested from living trees.^3^ Current commercial uses of turpentine include its use as a biofuel for power generation, its conversion into solvents and cleaning products (e.g., α-terpineol, camphene), and its fractional distillation into individual monoterpene components that are then used to produce flavors/fragrances (e.g., camphor, menthol), vitamins (e.g., vitamins E and D), and antioxidants (e.g., β-carotene).^2^ These transformations can potentially be used as the basis of a terpene-based biorefinery, so the development of scalable processes to produce additional biorenewable products from bulk monoterpene feedstocks is highly desirable.^4−7^ We have recently reported an optimal acid catalyzed ring opening process (6 M H_2_SO_4_, 90 °C, 2–4 h) to convert the major bicyclic monoterpene components (α-pinene, β-pinene, and 3-carene) present in the bulk monoterpene feedstocks CST, turpentine, and eucalyptus oil (cineole) into thermodynamic mixtures of *p*-menthadienes (α-terpinene, γ-terpinene, and isoterpinolene) (*p*-MeDs) (Figure 1).^8^ Importantly, significant quantities of other biorenewable *p*-MeD feedstocks are also available as byproducts of acid catalyzed hydrolysis processes used to convert turpentine into α-terpineol and camphene, while ∼30 000 t of limonene (a *p*-MeD) is available as a waste product from fruit peel generated by the citrus industry.^9−13^ The synthetic utility of these *p*-MeD mixtures as biorenewable substrates in ozonolysis, Diels–Alder reactions, and hydrogenation reactions^8,14^ has been demonstrated to produce biorenewable intermediates that are useful for the production of fragrance precursors, antioxidants, drug precursors, biofuels, solvents, and biopolymers (Figure 1).^4,15−19^

**Figure 2:**
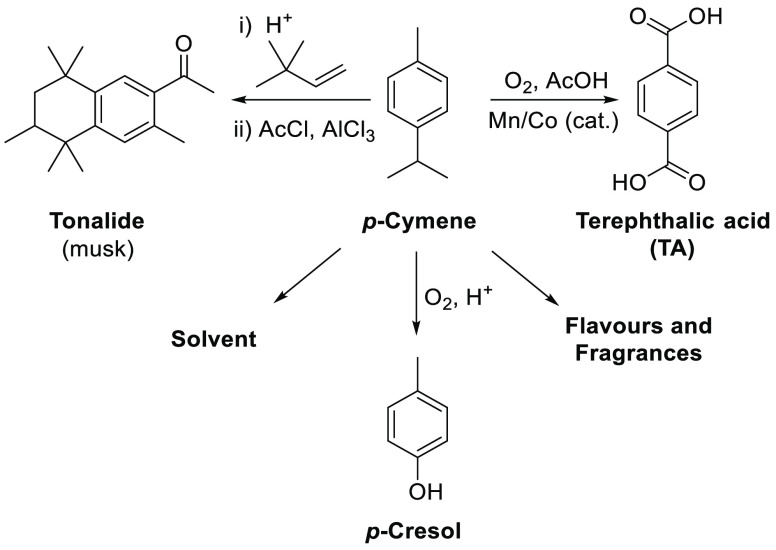
Use of *p*-cymene as a solvent and as a synthetic intermediate for chemical production.^8,14^

A potential advantage of using monoterpene-based feedstocks for biorenewable chemical synthesis is the ability to convert their preexisting *p*-menthene ring systems into the wide range of aromatic products that are currently sourced from petroleum feedstocks.^8,20,21^ Therefore, a key transformation developed during this valorization study was the oxidative isomerization of *p*-MeD mixtures into synthetically useful bio-*p*-cymene in 65–75% yields (Figure 1).^14^ This isoaromatization process could be achieved in batch reactions using 5 mol % Me_2_S, 1 equiv of *p*-cymene, O_2_, and 100 °C,^8,14^ or in flow reactions using 1 equiv of ^t^BuOOH, 2 equiv of *p*-cymene, O_2_, and 138 °C.^22^*p*-Cymene is a useful aromatic intermediate that is produced industrially on a kilotonne scale through Friedel–Crafts alkylation of petrochemically sourced toluene, with mixtures of cymene regioisomers then separated using the energy intensive Cymex process.^23^ Major commercial uses of *p*-cymene include its use for the production of tonalide (synthetic musk) and other fragrances, for the synthesis of *p*-cresol (antioxidant precursor), and as a green solvent for cleaning applications (Figure 2).^8,14^ Therefore, the availability of facile catalytic routes from multiple terpene feedstocks to bio-*p*-cymene would provide convenient pathways to a series of commercially important products that are currently sourced from nonrenewable petrochemical sources.

**Figure 3:**
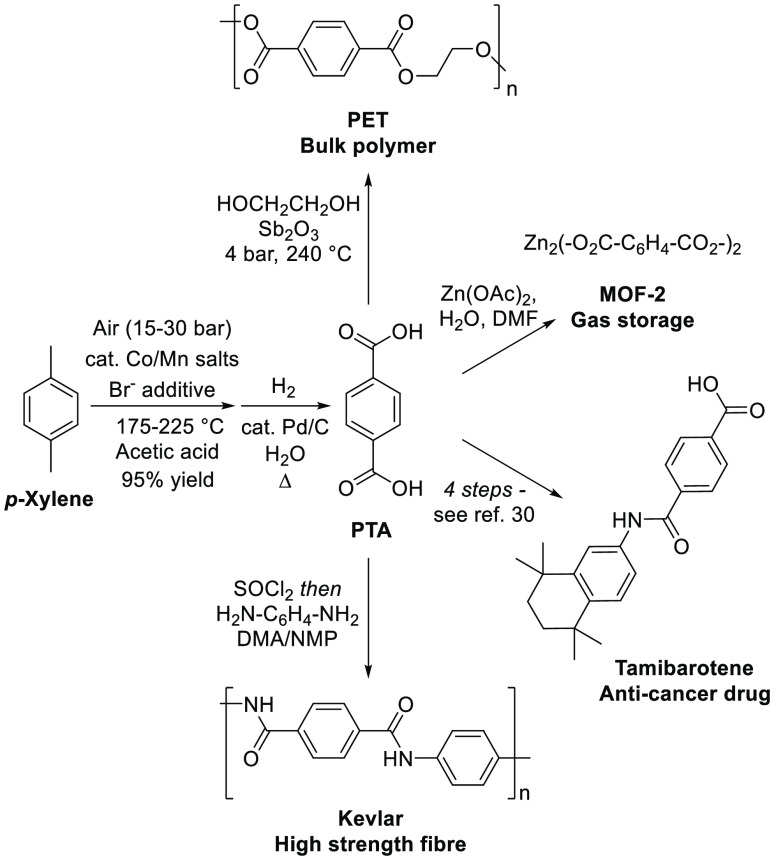
Industrial Amoco process used to convert *p*-xylene into PTA, which is useful as a bulk chemical feedstock for chemical and polymer production.^28^

Another potentially attractive commercial application for bio-*p*-cymene is its use as a drop-in bioreplacement for petroleum derived *p*-xylene that is currently used to produce the bulk commodity chemical terephthalic acid (TA).^24^ The industrial Amoco process used to transform *p*-xylene into TA involves use of a mixed Co(OAc)_2_/Mn(OAc)_2_ catalyst and a bromide promoter (commonly referred to as the “Mid-Century Catalyst”) in acetic acid under 15–30 bar air at temperatures between 175 and 225 °C.^25^ Purification of crude TA by Pd/C catalyzed, high pressure, aqueous phase hydrogenolysis is then used to remove the undesirable impurity 4-carboxybenzaldehyde (4-CBA) to afford “polymer-grade” purified terephthalic acid (PTA) that is then condensed with ethylene glycol to afford polyethylene terephthalate (PET).^25^ Global consumption of PTA was estimated to be >65 million tons in 2018, with this figure predicted to grow by around 6% annually for the foreseeable future.^24,26^ Most of the PTA produced is condensed with ethylene glycol to prepare PET as a bulk polymer for the production of synthetic fibers (e.g., for fabrics) and clear plastic packaging (e.g., for water/soda bottles).^24^ However, smaller amounts of PTA are also used for production of lower volume, high-value products, including high-performance polymers (e.g., Kevlar), metal–organic frameworks (e.g., for gas storage), and various drug molecules (e.g., tamibarotene).^27−31^ Therefore, development of a variant of the Amoco process that could be used to catalytically convert bio-*p*-cymene into bio-PTA would increase the availability of biorenewable chemicals/polymers from bulk terpene feedstocks (Figure 3).

Consequently, this study now describes our investigations into developing an industrially compatible variant of the Co^2+^/Mn^2+^ catalyzed Amoco process that can be used to oxidize the isopropyl and methyl side chains of bio-*p*-cymene into bio-TA in good yield. The elevated-pressure aerobic process that we have identified employs a cheap mixed Co(NO_3_)_2_/MnBr_2_ catalyst system (both < $0.20/g) to convert bio-*p*-cymene into highly crystalline bio-TA (30 bar air, acetic acid, 125 °C, 6 h) in ∼70% yields. The catalytic metal salts present in the mother liquors of spent *p*-cymene oxidation reactions can be recycled in serial bio-*p*-cymene oxidation reactions with no noticeable losses in catalytic activity or TA yield between each run. Furthermore, carrying out catalytic oxidation reactions of *p*-cymene under milder oxidative conditions using mixed Co(OAc)_2_/MnBr_2_ (1 atm air, 120 °C, 24 h) or Co(NO_3_)_2_/MnBr_2_ (1 atm O_2_, 120 °C, 24 h) catalytic systems can be used to selectively produce *p*-methylacetophenone (*p*-MA) or *p*-toluic acid as major products in 55–65% yields, respectively. Therefore, these new catalytic aerobic processes significantly broaden the range of biorenewable aromatic products that can be produced from bio-*p*-cymene within a terpene based biorefinery.

## Results and Discussion

With viable routes to transform biorenewable bulk terpene feedstocks into bio-*p*-cymene established, we wanted to identify optimal catalytic aerobic conditions to convert it into bio-TA (and hence bio-PET).^32^ Ideally, we aimed to identify conditions that were compatible with industrial Amoco processes [e.g., 1 mol % Co(OAc)_2_/Mn(OAc)_2_, 1 mol % bromide additives (e.g., HBr, NH_4_Br, NaBr), 15–30 bar air at 175–225 °C in AcOH)] that are used to oxidize petrochemically derived *p*-xylene into PTA in 95–99% yields on a megaton scale.^25^ This would allow bio-*p*-cymene to be used as a direct “drop-in” biorenewable replacement for *p*-xylene in industrial processes used to prepare PTA on a tonne scale in large chemical plants.^24^ These *p*-xylene oxidation plants would invariably require some modifications to enable bio-*p*-cymene to be used as a feedstock. However, use of similar catalyst/solvent systems should ensure good compatibility with existing reactors. The benefits of using the Amoco process to transform *p*-xylene into PTA are well-established, including reduced metal catalyst loadings, use of bromide salts/additives to generate bromine radical species that initiate efficient benzylic hydrogen atom abstraction reactions,^33^ synergistic action of Co/Mn cocatalysts to regenerate bromine radicals, and minimization of competing reaction pathways that can lead to byproducts and oxidative degradation of acetic acid solvent.^25^ However, the presence of the isopropyl group of *p*-cymene also has the potential to cause problems in Amoco-type processes, with competing oxidative processes potentially affording phenolic byproducts that can inhibit oxidative radical processes, formation of brominated aryl byproducts that undergo hydrodebromination during the TA purification process, and the detrimental effects of acetaldehyde/formic acid cleavage products on oxidative radical processes.^25^ However, development of controllable catalytic aerobic conditions that would allow selective oxidation of the isopropyl group of bio-*p*-cymene had the advantage of potentially affording bio-*p*-MA and bio-*p*-toluic acid as alternative biorenewable products.

A review of the literature revealed two promising patents describing Amoco-like conditions that employed mixed Co/Mn catalytic systems in the presence of bromide additives for the elevated-pressure aerobic oxidation of *p*-cymene into TA. The first report described that treatment of *p*-cymene with a mixed 0.5% Co(OAc)_2_/0.25% Mn(OAc)_2_ catalytic system in the presence of 0.5% NaBr in propionic acid under 1 bar O_2_ at 130 °C for 30 h gave TA in 67% yield.^34^ Alternatively, the second report described that use of a mixed Co(OAc)_2_/Mn(OAc)_2_ catalytic system in the presence of 0.5% LiBr additive in acetic acid under 15 bar air at 180 °C for 5 h gave TA in 65% yield.^34,35^ Therefore, we used these promising mixed metal catalyst/bromide additive conditions to guide our initial choice of conditions directed toward identifying optimal Amoco-like conditions for the catalytic aerobic conversion of bio-*p*-cymene into bio-*p*-MA, biotoluic acid, and bio-TA, respectively.

### Catalytic Atmospheric-Pressure Aerobic Oxidation of *p*-Cymene into *p*-MA and *p*-Toluic Acid

Initial studies commenced with an investigation into developing mild catalytic aerobic conditions to transform bio-*p*-cymene into bio-*p*-MA in good yield. Treatment of *p*-cymene with Co(OAc)_2_ (2.5 mol %)/Mn(OAc)_2_ (2.5 mol %) in acetic acid at 120 °C under 1 atm O_2_ for 48 h gave *p*-MA as the major product in 20% yield (Table 1, entry 1). Inclusion of NaBr (5 mol %) as an additive in this catalytic aerobic reaction resulted in an increase in *p*-MA yield from 20 to 42% (Table 1, entry 2), consistent with the ability of bromine radical species to promote benzylic hydrogen abstraction processes.^25^ Amoco processes used to transform *p*-xylene into PTA are normally carried out in the presence of bromide additives;^25^ however, we reasoned that bromine radical species could also be generated through the use of a metal bromide catalyst. Consequently, *p*-cymene was refluxed with CoBr_2_ (2.5 mol %)/Mn(OAc)_2_ (2.5 mol %) in acetic acid at 120 °C in air for 24 h, which gave *p*-MA as a major product in 55–60% yield, as well as small amounts of recovered *p*-cymene (∼5%) and *p*-toluic acid (10%) (Table 1, entry 3). Fractional distillation of the crude reaction product (bp values: *p*-cymene = 177 °C; *p*-MA = 226 °C; *p*-toluic acid = 274 °C) obtained from this catalytic aerobic reaction enabled pure *p*-MA to be reproducibly isolated in 55–65% yields.

**Table 1:**
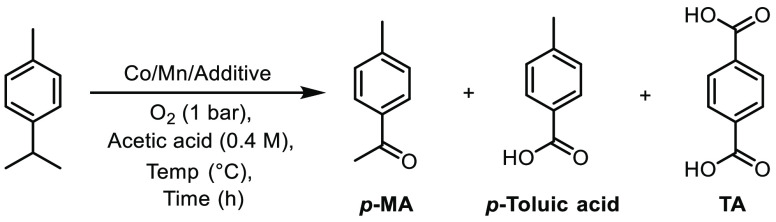
Optimization of Atmospheric-Pressure Conditions Used for Co(II)/Mn(II) Catalyzed Oxidation of *p*-Cymene into *p*-MA, *p*-Toluic Acid, and Terephthalic Acid

entry	temp (°C)	time (h)	Co source (2.5 mol %)	Mn source (2.5 mol %)	additive (mol %)	yield of *p*-MA (%)	yield of *p*-toluic acid (%)	yield of TA (%)
1	120	48	Co(OAc)_2_	Mn(OAc)_2_	–	20	2	0
2	120	48	Co(OAc)_2_	Mn(OAc)_2_	NaBr (5%)	42	5	0
3^a^	120	24	CoBr_2_	Mn(OAc)_2_	–	**60**	10	0
4	120	24	Co(NO_3_)_2_	Mn(NO_3_)_2_	–	12	27	0
5	120	24	CoBr_2_	Mn(NO_3_)_2_	–	26	48	2
6	120	24	Co(NO_3_)_2_	MnBr_2_	–	17	**56**	7
7	100	24	Co(NO_3_)_2_	MnBr_2_	–	16	54	12
8	100	48	Co(NO_3_)_2_	MnBr_2_	–	9	20	**49**

a Reaction carried out in air.

Our attention then turned to identifying catalytic aerobic conditions to oxidize *p*-cymene into *p*-toluic acid under more forcing oxidative conditions. A review of the literature revealed that metal nitrate salts had been reported to be more effective than their corresponding acetate salts in mixed metal catalytic systems used to oxidize acetophenones into their corresponding benzoic acids.^36^ This was consistent with previous reports that highly oxidizing nitrate radicals are efficient at abstracting tertiary benzylic hydrogen atoms in other catalytic benzylic oxidation reactions.^37−40^ Consequently, we decided to incorporate a nitrate counterion into the mixed metal catalyst system used to oxidize *p*-cymene, with the hope that any nitrate radicals produced would serve to generate higher yields of *p*-toluic acid. Use of a mixed Co(NO_3_)_2_/Mn(NO_3_)_2_ catalyst at 120 °C for 24 h was successful in producing a greater 27% yield of *p*-toluic acid, along with a 12% yield of *p*-MA (Table 1, entry 4). It had previously been reported that mixtures of cobalt bromide/manganese nitrate salts in acetic acid produced bromine radicals *in situ*,^41^ so we decided to trial use of a mixed metal nitrate/metal bromide catalytic system to see whether improved yields of *p*-toluic acid would be obtained, with use of a mixed CoBr_2_/Mn(NO_3_)_2_ catalytic system at 120 °C leading to *p*-toluic acid in an increased 48% yield (Table 1, entry 5). Switching the bromide and nitrate counterions of the metal catalysts resulted in a mixed Co(NO_3_)_2_/MnBr_2_ catalyst at 120 °C producing *p*-toluic acid in an even better 56% yield (Table 1, entry 6). Repeating the catalytic aerobic reaction using a mixed Co(NO_3_)_2_/MnBr_2_ catalyst at a slightly lower temperature of 100 °C for 24 h produced *p*-toluic acid in 54% yield, with a 12% yield of fully oxidized TA being observed for the first time (Table 1, entry 7). We propose that the increased solubility of O_2_ in acetic acid at 100 °C may be responsible for the combined 66% yield of *p*-toluic acid (54%) and TA (12%) being greater than the 56 and 7% yields of *p*-toluic acid and TA produced under otherwise identical conditions at 120 °C. Alternatively, oxidation of *p*-cymene at higher temperatures could potentially lead to substrate/solvent degradation.^42^ A brief optimization study identified that treatment of *p*-cymene with a Co(NO_3_)_2_ (2.5 mol %)/MnBr_2_ (2.5 mol %) catalytic system in acetic acid under 1 atm O_2_ at 120 °C for 24 h gave *p*-toluic acid in 55–60% yield, along with minor amounts of *p*-MA (∼20%) and TA (<5%). The crude product was easily purified through flash distillation of the acetic acid solvent, base extraction (NaHCO_3(aq)_), acidification (6 M H_2_SO_4_), extraction with EtOAc, and solvent removal to afford *p*-toluic acid in 60% yield.

Finally, running the Co(NO_3_)_2_/MnBr_2_ catalyzed reaction at 100 °C for 48 h was found to produce TA as the major product in a promising 49% yield, along with a 20% yield of *p*-toluic acid and a 9% yield of *p*-MA (Table 1, entry 8), with the white crystalline TA produced easily isolated by filtering the crude reaction mixture at room temperature. These atmospheric-pressure oxidation conditions for the production of TA from *p*-cymene are notably milder (100 °C, 1 bar O_2_) than those previously reported.^34^ This is likely to be due to use of a mixed Co(NO_3_)_2_/MnBr_2_ catalytic system that can generate mixtures of highly oxidizing nitrate and bromine radicals that can act synergistically to carry out more efficient benzylic hydrogen abstraction reactions.

**Figure 4:**
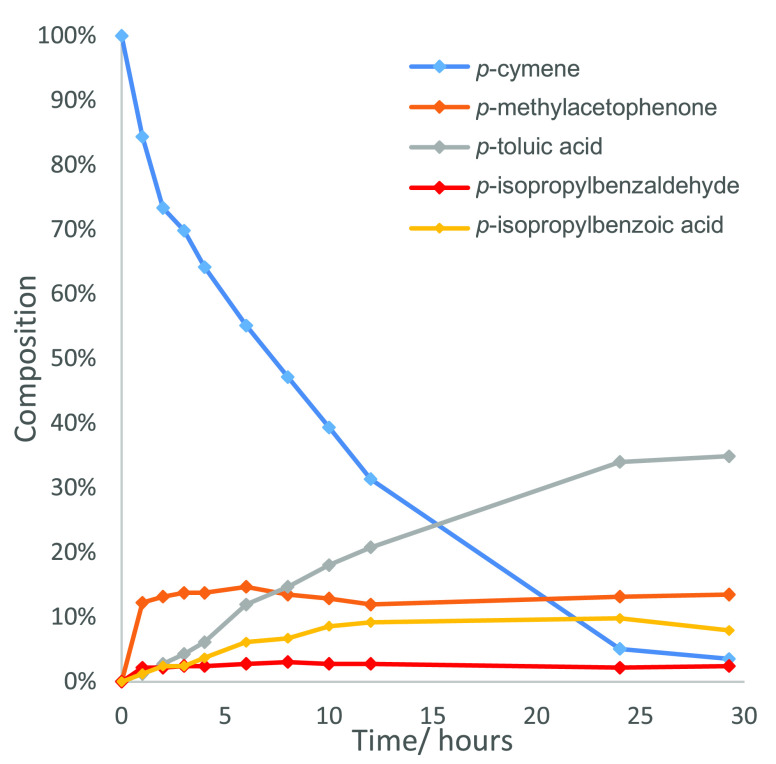
Reaction profile of the catalytic oxidation reaction of *p*-cymene to TA over time. Reaction conditions: *p*-cymene (1 equiv), Co(NO_3_)_2_ (2.5 mol %)/MnBr_2_ (2.5 mol %), AcOH (40 equiv), 1 atm O_2_, 100 °C.

^1^H NMR spectroscopic analysis of the mother liquor of a catalytic oxidation reaction of *p*-cymene (Co(NO_3_)_2_ (2.5 mol %)/MnBr_2_ (2.5 mol %), AcOH, 1 atm O_2_, 100 °C) over time revealed that 95% of the *p*-cymene was consumed after the first 24 h (Figure 4), with essentially no TA produced during this period (either in solution or as a precipitate). The fact that ∼4% *p*-cymene was still present after 30 h indicates that initial oxidation of the benzylic position of the isopropyl group is a relatively slow process. The concentration of *p*-MA increased to around 10–15% after ∼1 h, remaining relatively constant at this level for the remaining 29 h, thus indicating that it is formed as quickly as it is consumed during this period. Subjecting *p*-MA to the standard oxidative conditions resulted in its clean conversion to TA after 24 h in 99% yield, thus providing further evidence that the initial initiation oxidation step from *p*-cymene to *p*-MA is rate determining. The concentration of *p*-toluic acid increased steadily to around 30% after 24 h (no TA formed), indicating that oxidation of its methyl group is relatively slow under these conditions, with significant amounts of crystalline TA only appearing after 24 h.^25,43^ Increasing reaction times beyond 48 h did not improve the overall yield of TA (49%), suggesting that competent free radical oxidation pathways responsible for oxidizing *p*-MA (∼10% byproduct) to TA are no longer operating after this time. Formation of volatile methyl bromide may lead to loss of bromide promoter through evaporation over extended reaction times, while buildup of significant amounts of water may also deactivate the catalyst system.^25^ Only a small amount (<5%) of *p*-isopropylbenzaldehyde was present during the first 24 h of the reaction, indicating that oxidation of the isopropyl group of *p*-cymene is preferred over its methyl group, which is consistent with the relative stabilities of the tertiary and primary radicals produced in their corresponding benzylic oxidation reactions.^44^

**Figure 5:**
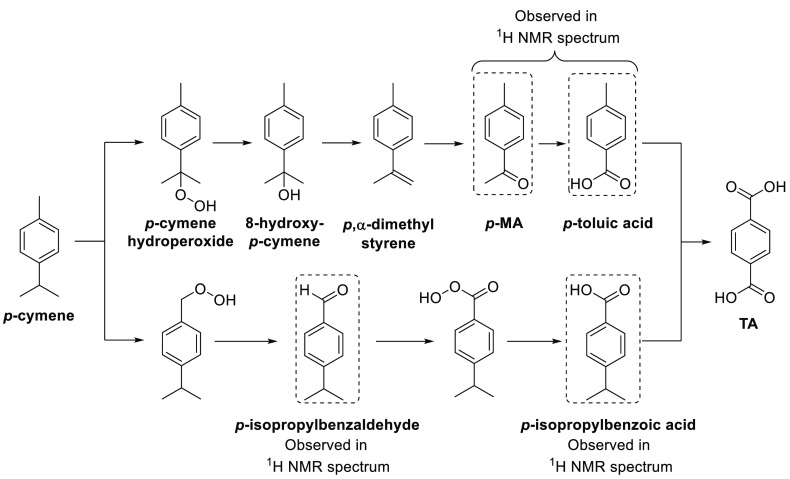
Major (top) and minor (bottom) oxidation pathways that occur in the catalytic aerobic oxidation of *p*-cymene into TA.

The flux of the different partially oxidized intermediates generated in the catalytic aerobic oxidation reaction of *p*-cymene over time is consistent with two oxidative pathways operating to produce TA (Figure 5). The major pathway involves initial slow oxidation of the benzylic position of the isopropyl group to afford *p*-cymene hydroperoxide, whose weak peroxy bond is then cleaved to afford 8-hydroxycymene (not observed) that then undergoes a formal dehydration reaction to produce *p*,α-dimethylstyrene (not observed). Further oxidation of the alkene bond of *p*,α-dimethylstyrene then produces the ketone group of *p*-MA (with loss of a formaldehyde equivalent), with further oxidation then occurring to produce *p*-toluic acid (with loss of a second formaldehyde equivalent). The methyl group of *p*-toluic acid then undergoes slow oxidation to afford the second carboxylic acid group of TA (via *p*-formylbenzoic acid), as occurs in Amoco oxidation reactions of *p*-xylene.^25^ The second minor oxidation pathway occurs through competing benzylic oxidation of the methyl group of *p*-cymene to afford *p*-isopropylbenzaldehyde (observed in the ^1^H NMR spectrum), which is then further oxidized to *p*-isopropylbenzoic acid (observed in the ^1^H NMR spectrum) whose isopropyl group is then converted into TA via a series of benzylic oxidation reactions similar to those that occur in the major pathway.

### Catalytic Elevated-Pressure Aerobic Oxidation of *p*-Cymene to TA

**Figure 6:**
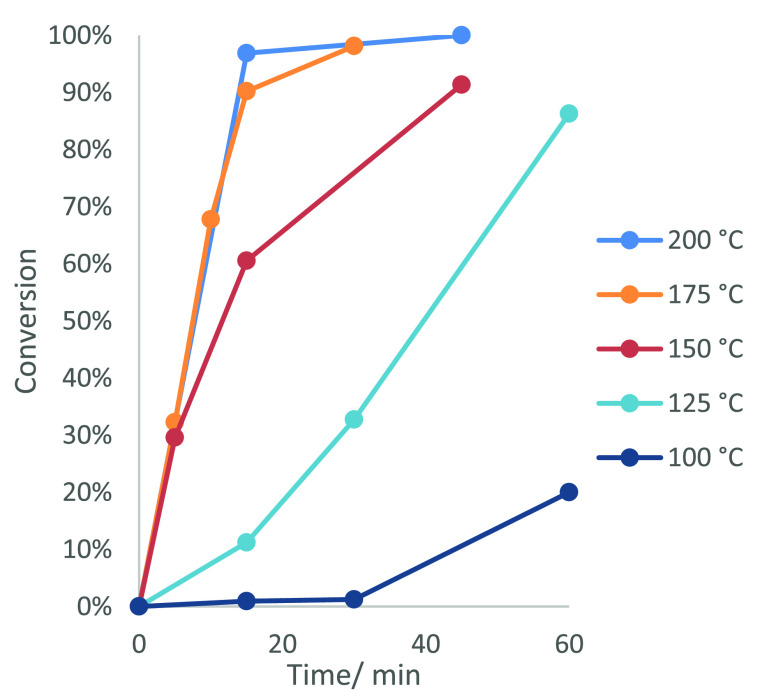
Rates of consumption of *p*-cymene using 2.5 mol % Co(NO_3_)_2_ and 2.5 mol % MnBr_2_ in acetic acid under 30 bar air at different temperatures between 100 and 200 °C.

Our attention then turned toward developing a catalytic elevated-pressure aerobic process for oxidizing *p*-cymene into TA in better yield. Elevated-pressure oxidation reactions of *p*-cymene were carried out using a semibatch glass-lined stainless steel autoclave with the capacity to flow an oxidizing gas through a pressurized solution of *p*-cymene in acetic acid at atmospheres up to 36.5 bar pressure of air (or 10% O_2_ in N_2_) (see Figures S1 and S2 for details). The gas inlet tube supplying the pressure reactor with oxygen was fitted with a gas-porous PTFE membrane to prevent it from becoming blocked with the highly crystalline TA product that precipitates from solution over the course of the *p*-cymene oxidation reaction (see the Supporting Information for details). Elevated-pressure oxidation reactions of *p*-cymene were carried out with a mixed 2.5 mol % Co(NO_3_)_2_/MnBr_2_ catalyst system in acetic acid under 30 bar air or 10% O_2_ in N_2_ at different temperatures. A significant increase in the rate of consumption of *p*-cymene was observed as the temperature was raised from 100 to 200 °C, with step changes in reactivity observed between 100 and 125 °C, between 125 and 150 °C, and between 150 and 200 °C, respectively (see Figure 6). For example, it took 1 h for 85% of the *p*-cymene to be consumed at 125 °C, >90% of the *p*-cymene was consumed after 45 min at 150 °C, and >98% of the *p*-cymene was consumed after 15 min at 175 °C.

**Figure 7:**
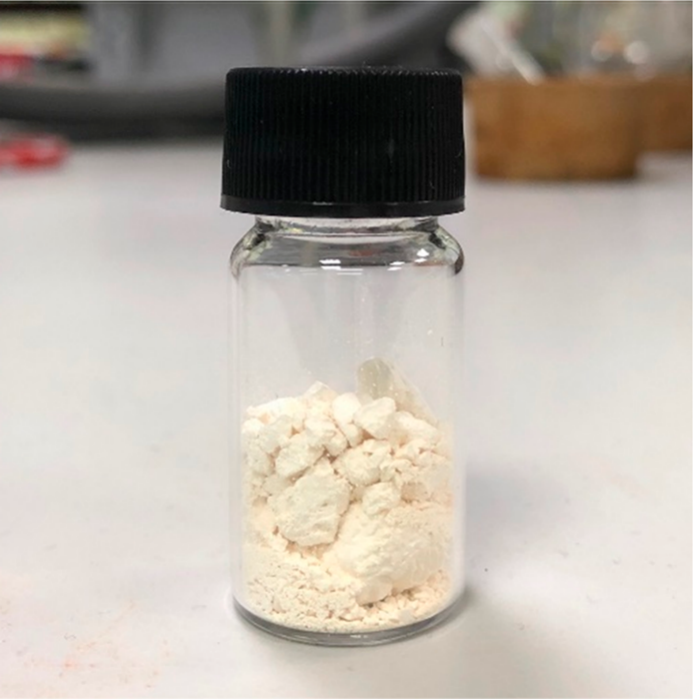
White crystalline TA obtained from filtration of an untreated crude reaction product produced in an optimal elevated-pressure *p*-cymene oxidation reaction at 125 °C.

Analysis of the products present in the elevated-pressure *p*-cymene oxidation reactions using 30 bar air (or 10% O_2_ in N_2_) at 100 °C revealed that no TA was present after 6 h (Table 2, entry 1); however, use of 30 bar 10% O_2_ in N_2_ at 150 °C produced a 62% yield of TA after 6 h (Table 2, entry 2). As expected for a reaction where oxygen mass transfer is rate limiting, switching to use of 30 bar air at 150 °C (higher O_2_ partial pressure, greater concentration of dissolved O_2_) gave further improvement, producing a better 70% yield of crystalline TA at 150 °C after 6 h (Table 2, entry 3). Elevated-pressure aerobic reactions of *p*-cymene using 30 bar air at 175 and 200 °C also produced similar 68–70% yields of TA (Table 2, entries 4 and 5). However, use of temperatures of ≥150 °C in these elevated-pressure aerobic reactions (Table 2, entries 3–5) produced light yellow crystalline TA products, with formation of colored TA in Amoco reactions of *p*-xylene known to be associated with the presence of dimeric byproducts (e.g., 2,6-dicarboxyanthraquinone and 2,6-dicarboxyfluorenone),^45^ partially oxidized intermediates (e.g., 4-formylbenzoic acid), or brominated byproducts (e.g., benzylic bromides).^42^ Formation of yellow TA is problematic due to transparent colorless PET polymer generally being required for commercial applications; however, PTA purification processes normally convert these yellow impurities into colorless products. Consequently, optimal conditions were established based on treatment of *p*-cymene with 2.5 mol % Co(NO_3_)_2_/2.5 mol % MnBr_2_ in acetic acid (40 equiv) under 30 bar air at 125 °C for 6 h (cf. 175–225 °C for 2–24 h in industrial Amoco processes of *p*-xylene), which reproducibly gave white crystalline TA in ∼70% yield (Table 2, entry 6). The TA product produced in this process could be isolated from the reactor by simply releasing the reactor pressure, cooling the mother liquor to room temperature, and then filtering off the highly crystalline TA (see Figure 7) from the mother liquor.

**Table 2:**
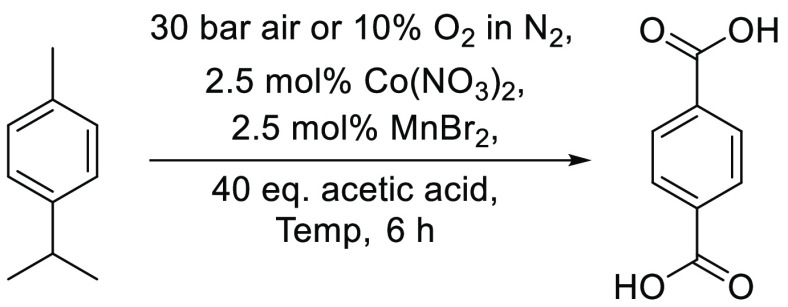
Optimization of the Elevated-Pressure Oxidation Reaction of *p*-Cymene to TA

entry	oxidizing gas	*T* (°C)	isolated yield (%)
1	air or 10% O_2_ in N_2_	100	0
2	10% O_2_ in N_2_	150	62
3	air	150	70
4	air	175	68
5	air	200	68
6	air	125	**70**

**Figure 8:**
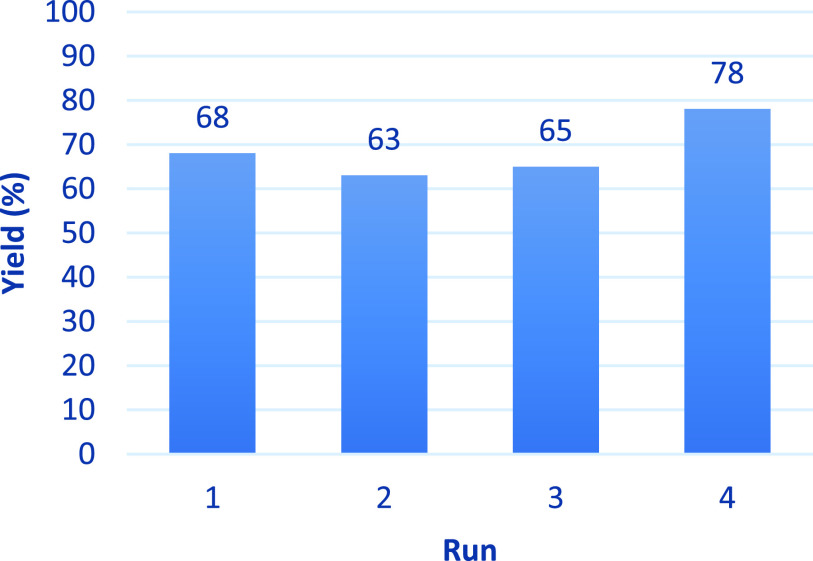
Yields of white crystalline TA (63–78%) obtained from recycling mother liquors in consecutive *p*-cymene oxidation reactions under optimal elevated-pressure aerobic conditions.

We next explored the possibility of increasing the efficiency and economic viability of this elevated-pressure oxidative process from *p*-cymene to TA by investigating whether we could recycle the metal catalysts, partially oxidized aromatic intermediates, and acetic acid (solvent) present in the mother liquor. ^1^H NMR spectroscopic analysis of the filtered mother liquor obtained from an optimal elevated-pressure *p*-cymene oxidation reaction (2.5 mol % Co(NO_3_)_2_/2.5 mol % MnBr_2_ in acetic acid, 30 bar air, 125 °C, 70% yield of TA) revealed that it contained useful quantities of *p*-MA (∼5%) and *p*-toluic acid (∼10%). Fresh *p*-cymene (1 equiv) was added to the mother liquor filtrate, and a small amount of acetic acid (<10%) was added to replenish the solvent volume (no new metal catalysts added). This reaction mixture was then used to carry out a new *p*-cymene oxidation reaction at 125 °C under 30 bar air to produce a second batch of crystalline TA in 63% yield after 6 h. The resultant mother liquor filtrate obtained from this second oxidation reaction was then recycled two further times to transform fresh *p*-cymene into third and fourth batches of TA in 65 and 78% yields, respectively (Figure 8), with no noticeable change in the quality of the TA product produced after each run. The increase in yields in later recycling experiments can be explained due to the presence of a greater concentration of the intermediates *p*-MA and *p*-toluic acid in the mother liquors of initial runs. Therefore, these recycling results demonstrate that the catalytic metal complex species present in the untreated mother liquors can be used at least four times with no significant losses in catalyst activity, which significantly enhances the economic/environmental credentials of this catalytic aerobic process.

For comparative purposes, our elevated-pressure experimental rig was then used to oxidize *p*-xylene using 2.5 mol % Co(NO_3_)_2_/2.5 mol % MnBr_2_ in acetic acid (40 equiv) at 200 °C under 30 bar air, which gave an 85% yield of white crystalline TA after 6 h. The conditions used to produce 90–95% yields of TA in optimal industrial elevated-pressure Amoco processes of *p*-xylene have been optimized over many years. Consequently, we anticipate that the ∼70% yields of TA produced from *p*-cymene in this pilot study could potentially be improved further in a modified and fully optimized industrial process. The optimal catalytic elevated-pressure aerobic conditions used to convert terpene derived bio-*p*-cymene into bio-TA in ∼70% yields compares favorably with other single-step catalytic aerobic routes that have been reported previously, with previous processes generally lower yielding, employing higher catalyst loadings, and/or requiring higher temperatures to proceed (see Table 3).^34,35,46−51^

**Table 3:**
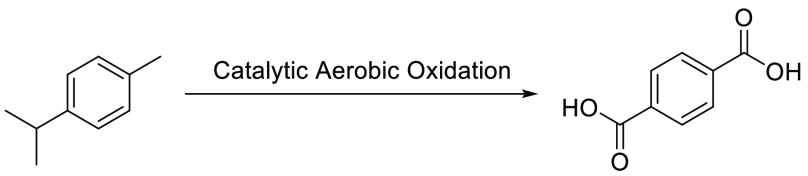
Single-Step Catalytic Aerobic Oxidation Conditions Used to Transform *p*-Cymene into TA

catalyst	conditions	yield (%)	ref
Mn/Fe/O mixed metal oxide (6 wt %)	140 °C, 20 bar O_2_, 24 h	51	(46)
V_2_O_5_ (1%)	200 °C, 0.2 bar air, 1 h, flow reactor	40	(47)
Co/Mn mixed metal oxide (6 wt %)	140 °C, 20 bar O_2_, 6 h	12	(48)
Fe_2_O_3_ (7%)	NaOH (3 equiv), H_2_O, sodium stearate (4%), 20 bar air, 160 °C	62	(49)
MnBr_2_ (1%)	acetic acid, 180 °C, 24 bar air, 2 h	55–70	(50)
Mn(OAc)_2_ (0.95%), NH_4_Br (0.75%)	acetic acid, 180 °C, 18 bar air, 2 h	59	(51)
Co(OAc)_2_ (0.5%), Mn(OAc)_2_ (0.25%)	propionic acid, 130 °C, 1 bar O_2_, 30 h	67	(34)
NaBr (0.5%)			
Co(OAc)_2_ (0.25%), Mn(OAc)_2_ (0.28%), LiBr (0.5%)	acetic acid, 180 °C, 15 bar air, 5 h	65	(35)

### Use of Bio-*p*-MA, Bio-*p*-toluic Acid, and Bio-PTA in a Terpene Biorefinery

**Figure 9:**
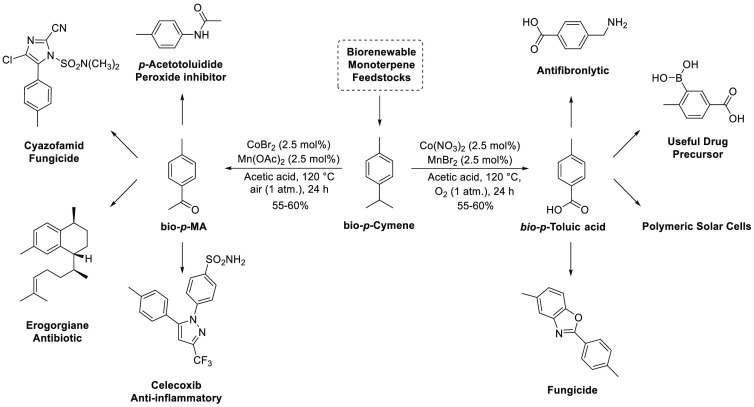
Range of biorenewable products available from bio-*p*-MA and bio-*p*-toluic acid.^52−57^

Successful economic production of biorenewable chemicals in a terpene biorefinery requires access to a range of reaction pathways that can be used to transform multiple biorenewable terpene feedstocks into different value-added product streams. Petroleum derived *p*-MA, *p*-toluic acid, and PTA are widely used as chemical intermediates for the industrial synthesis of a wide range of commercially available products (e.g., perfumes, drugs, inks, polymers, solar cells, antioxidants, and MOFs).^52−57^ Therefore, use of scalable catalytic aerobic transformations to convert terpene derived bio-*p*-cymene into bio-*p*-MA, bio-*p*-toluic acid, and bio-PTA is particularly useful for increasing the range of aromatic bioproducts that can be produced in a terpene biorefinery (Figure 9).

Polymerization of terpene derived bio-PTA with biorenewable ethylene glycol (already produced from lignocellulose biomass^24^) would enable access to fully biorenewable PET for the production of synthetic fibers (e.g., for clothing) and clear plastic packaging (e.g., to produce water/soda bottles). The global annual demand for PTA (produced from petrochemically derived *p*-xylene) for PET production is >65 million t,^24,25^ which dwarfs the ∼400 000 t annual commercial volumes of biorenewable terpene feedstocks (CST ∼ 260 000 t year^–1^; GT ∼ 100 000 t year^–1^; limonene ∼30 000 t year^–1^; 1,8-cineole ∼7000 t year^–1^).^8,20^ Therefore, any bio-PTA produced by a terpene biorefinery for bio-PET production is likely to be used for the production of premium “green” products (e.g., fabrics for high-end fashion items, plastic containers for designer cosmetics), with any additional cost associated with bio-PET use offset by the ability of companies to market a fully biorenewable product. Looking to the future, introduction of environmental legislation (e.g., from the European Union) is likely to drive the transition from nonsustainable plastics to the use of fully biorenewable polymers, which should increase the future cost competitiveness of bio-PET.^58^ Biotechnological developments have recently shown that recombinant microorganisms and cell-free biocatalytic systems can be used to ferment cheap abundant lignocellulosic biomass into monoterpene products, which could potentially be useful for increasing future terpene feedstock volumes.^59−62^ This would enable large scale, geographically flexible terpene based biorefineries to be established to meet large scale demand for bio-PTA (and hence bio-PET) production in countries that do not have access to large arboreal resources. Bio-PTA is also potentially useful for the synthesis of a wide range of other higher value, lower volume products (see Figure 3), thus providing alternative biorenewable product streams to underpin the economics of bio-PTA production in a terpene biorefinery. The elevated-pressure catalytic aerobic protocols described herein complete the three-step route required to convert the bulk terpene feedstocks CST, GT and 1,8-cineole into bio-TA in 35–45% overall yields and a two-step route to convert limonene into bio-TA in 42% yield (see Figure 1 and Table 2). The overall yields of bio-TA produced in these monoterpene based routes compare favorably with and complement other pathways that have been developed to transform other types of biomass into bio-TA (see Figures S3 and S4 for details), although some of the lignocellulose based feedstocks used in these studies are potentially more abundant (and hence cheaper) than the monoterpene based feedstocks used in this study.^24,63−67^

## Conclusions

This study describes the development of a series of catalytic aerobic oxidation reactions of terpene derived bio-*p*-cymene that can be used to produce a series of oxygenated aromatics for the production of a range of biorenewable commercial products that are currently sourced from petrochemical precursors. These transformations are variants of well-established Amoco oxidative processes that are currently used to convert petrochemically derived *p*-xylene into PTA on a megaton scale. Use of a mixed CoBr_2_/Mn(OAc)_2_ catalytic system at 120 °C for 24 h enables partial oxidation of the isopropyl unit of *p*-cymene to produce *p*-MA in 55–60% yield. Alternatively, use of Co(NO_3_)_2_/MnBr_2_ under 1 atm O_2_ at 100 °C for 24 h affords *p*-toluic acid in 55–60% yield, with bromine and nitrate radicals proposed to act synergistically to carry out efficient benzylic hydrogen abstraction reactions that generate strong oxidative conditions under milder reaction conditions. Use of optimal elevated-pressure catalytic oxidative process (2.5 mol % Co(NO_3_)_2_, 2.5 mol % MnBr_2_, air (30 bar), 125 °C, acetic acid, 6 h) results in oxidation of *p*-cymene into crystalline white TA that can be isolated through simple filtration of the mother liquors in ∼70% yield. The metal catalysts present in the mother liquors of these elevated-pressure reactions could be recycled up to three times with no loss in catalytic activity or yield of TA (∼70%), thus improving the overall sustainability and economic viability of the process. Scalable catalytic routes can now be used to convert multiple biorenewable bulk terpene feedstocks (e.g., crude sulfate turpentine, gum turpentine, 1,8-cineole, and limonene) into synthetically useful bio-*p*-MA, bio-*p*-toluic acid, and bio-TA (and hence bio-PET), which significantly diversifies the range of biorenewable products available in a terpene based biorefinery.

## Experimental Section

### General Experimental Details

All reagents were purchased from commercial suppliers. Bio-*p*-cymene was prepared in 50–60% yield from CST using our previously reported two-step process,^8^ with CST obtained from a Swedish paper mill owned by Södra Forestry. All reactions were carried out in air unless otherwise stated. Nuclear magnetic resonance spectra were recorded with a Bruker Avance 300, 400, or 500 MHz or an Agilent Technologies 500 MHz spectrometer. All ^13^C spectra were run proton decoupled in CDCl_3_. Chemical shifts (δ) are reported in parts per million (ppm) and are referenced to the residual solvent peaks. Coupling constants (*J*) are quoted to the nearest 0.1 Hz. Abbreviations used in reporting peaks were s, br s, d, and m, to denote singlet, broad singlet, doublet, and multiplet, respectively.

### *p*-Methylacetophenone (*p*-MA)

*p*-Cymene (0.268 g, 2 mmol) was added to a solution of cobalt(II) bromide (0.011 g, 0.05 mmol) and manganese(II) acetate (0.009 g, 0.05 mmol) in glacial acetic acid (5 mL), and the stirred reaction mixture was heated at reflux for 24 h. The reaction mixture was cooled to room temperature, and the solvent was removed *in vacuo*. Saturated NaHCO_3_ (10 mL) was added to the crude product, which was then extracted with diethyl ether (10 mL). The organic extract was dried (MgSO_4_), and the solvent was removed *in vacuo* to afford a crude product that was purified by fractional distillation (bp 226 °C) to afford the title compound as a pale-yellow oil (0.155 g, 1.16 mmol, 58%).

^1^H NMR (500 MHz, CDCl_3_, δ): 7.86 (d, *J* = 7 Hz, 2H, Ar*H*), 7.26 (d, *J* = 7 Hz, 2H, Ar*H*), 2.58 (s, 3H, COC*H*_3_), 2.43 (s, 3H, ArC*H*_3_).

^13^C NMR (125 MHz, CDCl_3_, δ): 198.0, 144.0, 134.9, 129.4, 128.6, 26.7, 21.8.^68^

### *p*-Toluic Acid

*p*-Cymene (0.268 g, 2 mmol) was added to a solution of cobalt(II) nitrate hexahydrate (0.015 g, 0.05 mmol) and manganese(II) bromide (0.011 g, 0.05 mmol) in glacial acetic acid (5 mL). The reaction mixture was heated to reflux with oxygen then bubbled through the solution for 15 min. The reaction was sealed under a balloon of oxygen and then stirred at reflux for 24 h. The reaction mixture was then cooled to room temperature and filtered before the solvent was removed *in vacuo*. Saturated NaHCO_3_ (10 mL) was then added and extracted with ethyl acetate (10 mL). The aqueous layer was acidified with 6 M sulfuric acid and then back extracted with ethyl acetate (10 mL). The organic layer was dried (MgSO_4_) and the solvent removed *in vacuo* to afford the title compound as a white solid (0.152 g, 1.12 mmol, 56%).

^1^H NMR (500 MHz, CDCl_3_, δ): 8.00 (d, *J* = 7.5 Hz, 2H, Ar*H*), 7.27 (m, *J* = 7.5 Hz, 2H, Ar*H*), 2.43 (s, 3H, C*H*_3_).

^13^C NMR (125 MHz, CDCl_3_, δ): 171.1, 144.7, 130.4, 129.4, 126.7, 21.9.^69^

### Terephthalic Acid

#### Method 1. Atmospheric-Pressure Oxidation Conditions for Oxidation of *p*-Cymene into TA

*p*-Cymene (0.268 g, 2.00 mmol), cobalt(II) nitrate hexahydrate (0.015 g, 0.05 mmol), and manganese(II) bromide (0.011 g, 0.05 mmol) were dissolved in glacial acetic acid (5 mL). The reaction mixture was heated to 100 °C, and oxygen was bubbled through the solution for 5 min. The reaction was sealed with a balloon of oxygen, and the reaction was stirred at 100 °C for 48 h before it was cooled to room temperature and the precipitate was filtered off. The resultant solid was washed with acetic acid to afford the title compound as a white crystalline solid (0.162 g, 0.98 mmol, 49%).

#### Method 2. Elevated-Pressure Oxidation Conditions for Oxidation of *p*-Cymene into TA

*p*-Cymene (2.68 g, 20 mmol), cobalt(II) nitrate hexahydrate (0.15 g, 0.50 mmol), and manganese(II) bromide (0.11 g, 0.50 mmol) were dissolved in glacial acetic acid (50 mL) and charged into a glass-lined high-pressure stainless steel reactor (see the Supporting Information for details). The headspace of the reactor was pressurized with air to 30 bar, and the reaction was heated to 125 °C. The sparger inlet fitted with a Teflon membrane was then opened, and oxidizing gas was bubbled through the reaction at 150 mL/min under 30 bar for 6 h. The reaction was then cooled to room temperature, and the pressure was reduced to atmospheric pressure. The resultant white precipitate was filtered off and washed with acetic acid before it was dried in air overnight to give the title compound as a white solid (2.32 g, 14 mmol) in 70% yield.

#### Method 3. Three-Step Conversion of Scandinavian CST into TA

##### Step 1

CST (12.0 mL, 10.44 g, 77 mmol) was stirred at 500 rpm at 90 °C. H_2_SO_4_ (2.4 mL, 6 *m* aq) was added in one portion, and the reaction was stirred at 90 °C for 4 h. Stirring was then stopped, and the organic and aqueous layers were allowed to cool and separate. The organic layer containing the desired *p*-MeDs mixture (α-terpinene, γ-terpinene, isoterpinolene) was then decanted off and used directly in the next isoaromatization step.

##### Step 2

*p*-Cymene (12.0 mL, 10.29 g, 77 mmol) and Me_2_S (0.28 mL, 0.24 g, 3.8 mmol) were added to the crude sulfurous mixture of *p*-MeDs (containing 25% terpene oligomers), and the resultant mixture was heated to 100 °C. A steady stream of O_2_ was bubbled through the stirred solution for 2 h. The O_2_ stream was stopped, the reaction was cooled to room temperature, and the mixture was then distilled under reduced pressure to afford *p*-cymene (bp = 70 °C at 10 mmHg) as a colorless liquid (15.74 g, 118 mmol) in 53% yield (allowing for 1 equiv of *p*-cymene used as solvent).

##### Step 3

The elevated-pressure procedure described in Method 2 was then used to transform the bio-*p*-cymene (2.68 g, 20 mmol) from step 2 into TA, which was obtained as a white solid (2.30 g, 14 mmol) in 70% yield (37% overall yield from CST).

^1^H NMR (500 MHz, DMSO-*d*_6_, δ): 13.30 (s, 2H, COO*H*), 8.04 (s, 4H, Ar*H*).

^13^C NMR (125 MHz, DMSO-*d*_6_, δ): 166.7, 134.5, 129.5.^63^

## Supporting Information Available

Diagrams of experimental setups, comparative literature methods, and NMR spectra of products (PDF)
