# Supplementary material for: Efficient Syntheses of Biobased Terephthalic Acid, p-Toluic Acid, and p-Methylacetophenone via One-Pot Catalytic Aerobic Oxidation of Monoterpene Derived Bio-p-cymene
Source: ACS Sustain Chem Eng. 2021 Jun 17;9(25):8642–52. doi: 10.1021/acssuschemeng.1c02605 (PMC8735764; doi:10.1021/acssuschemeng.1c02605)
Supplement: Supplementary file 1 — sc1c02605_si_001.pdf [file sc1c02605_si_001.pdf]

Supporting Information

Efficient Syntheses of Biobased Terephthalic acid, *p*-Toluic Acid and *p*-Methylacetophenone via One-Pot Catalytic Aerobic Oxidation of Monoterpene Derived bio-*p*-Cymene

*Joshua D. Tibbetts,[a,b] Danilo Russo,[c] Alexei A. Lapkin[c] and Steven D. Bull[a]**

[a]Department of Chemistry, University of Bath, Claverton Down, Bath, BA2 7AY, UK

[b]Centre for Sustainable Chemical Technologies, University of Bath, Claverton Down, Bath, BA2 7AY, UK

[c]Department of Chemical Engineering and Biotechnology, University of Cambridge, West Cambridge Site, Philippa Fawcett Drive, Cambridge CB3 0AS, United Kingdom

E-mail: s.d.bull@bath.ac.uk

Number of pages: 10

Number of figures: 10

Number of tables: 0

Contents

Figure S1. Diagram of elevated pressure oxidation apparatus used for the oxidation of *p*-cymene to terephthalic acid 3

Figure S2. Photograph of elevated pressure oxidation apparatus used for the oxidation of *p*-cymene to terephthalic acid 4

Figure S3. Synthetic routes to *p*-toluic acid intermediate from biorenewable non-monoterpene feedstocks;, using: a) isoprene/acrylic acid; (b) isoprene/methane-derived propiolic acid; c) ethyl sorbate; and d) sugar-derived coumalic acid. 5

Figure S4. Synthetic routes to *p*-xylene or terephthalic acid from lignocellulosic biorenewable feedstocks;, using: a) 5-hydroxymethylfurfural (5-HMF);, b) bio-isobutanol; and c) muconic acids. ..6

Figure S5. ^{1}H NMR spectrum of *p*-methylacetophenone. 7

Figure S6. ^{13}C NMR spectrum of *p*-methylacetophenone. 7

Figure S7. ^{1}H NMR spectrum of *p*-toluic acid. 8

Figure S8. ^{13}C NMR spectrum of *p*-toluic acid. 8

Figure S9. ^{1}H NMR spectrum of terephthalic acid. 9

Figure S10. ^{13}C NMR spectrum of terephthalic acid. 9

References 10

Figure S1. Diagram of elevated pressure oxidation apparatus used for the oxidation of *p*-cymene to terephthalic acid

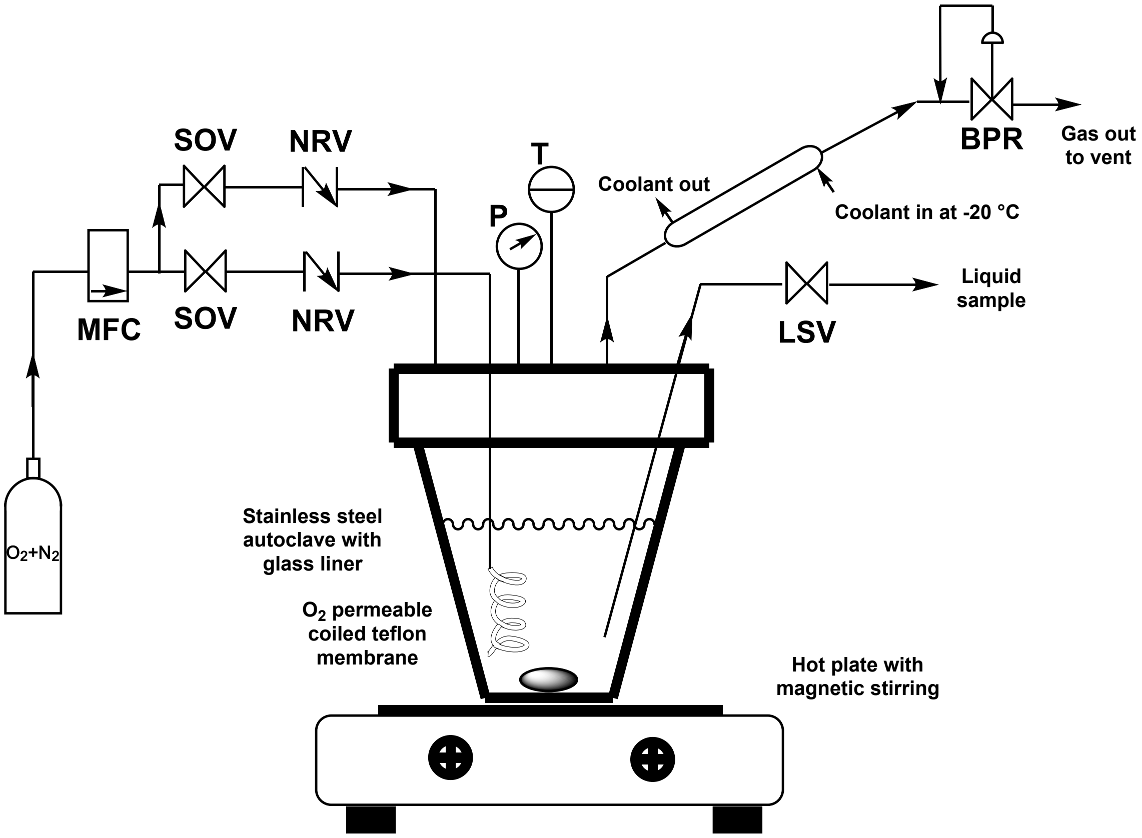

MFC = Mass flow controller

SOV = Shut off valve

NRV = Non return valve

LSV = Liquid sampling valve

BPR = Back pressure regulator

T = Temperature probe

P = Pressure gauge

Figure S2. Photograph of elevated pressure oxidation apparatus used for the oxidation of *p*-cymene to terephthalic acid

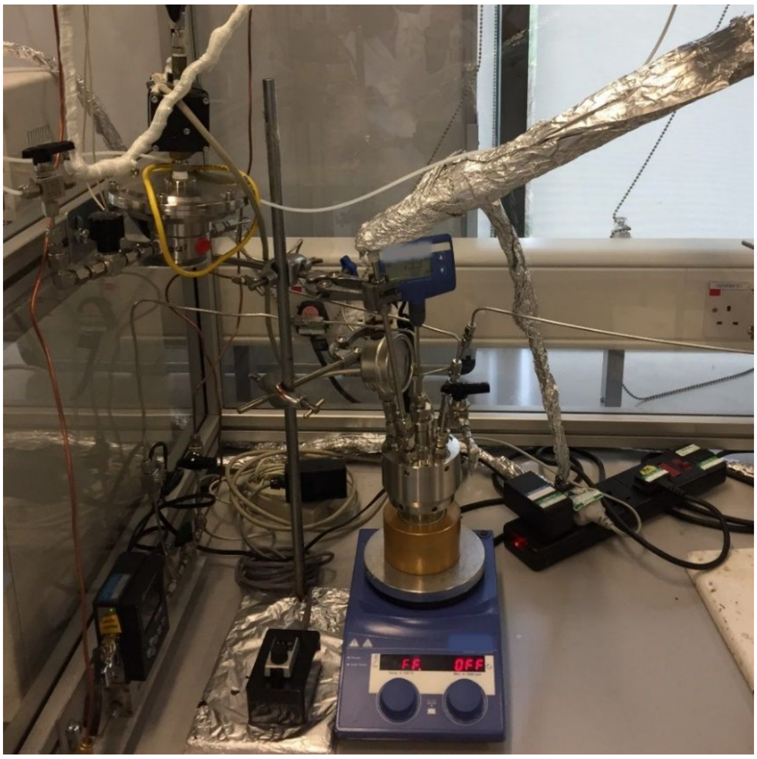

Figure S3. Synthetic routes to *p*-toluic acid intermediate from biorenewable non-monoterpene feedstocks;[1,2] using: a) isoprene/acrylic acid;[3] (b) isoprene/methane-derived propiolic acid;[4] c) ethyl sorbate;[5] and d) sugar-derived coumalic acid.[6]

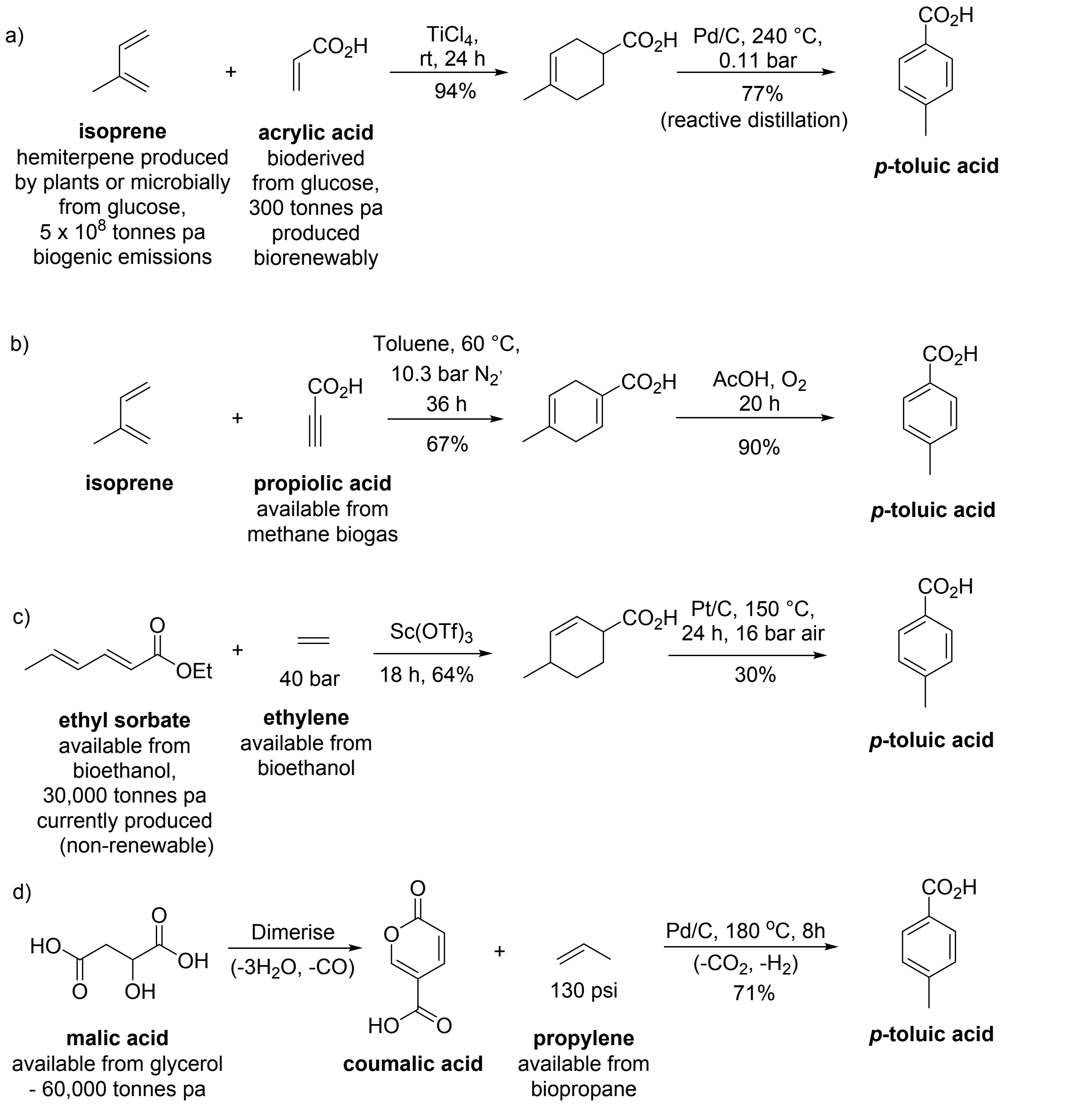

Figure S4. Synthetic routes to *p*-xylene or terephthalic acid from lignocellulosic biorenewable feedstocks;[2,7] using: a) 5-hydroxymethylfurfural (5-HMF);[8,9] b) bio-isobutanol;[10] and c) muconic acids.[11]

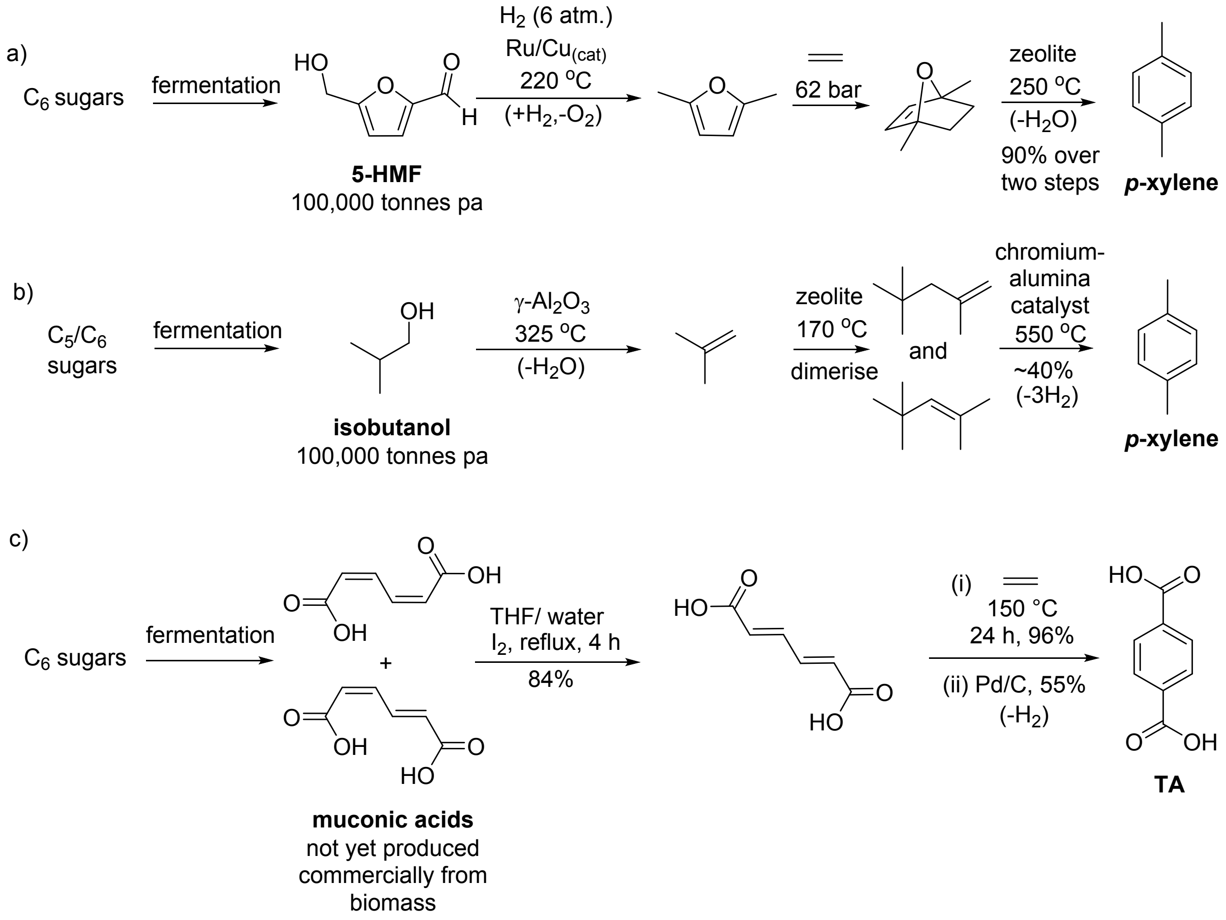

^{1}H and ^{13}C NMR spectra of products

Figure S5. ^{1}H NMR spectrum of *p*-methylacetophenone.

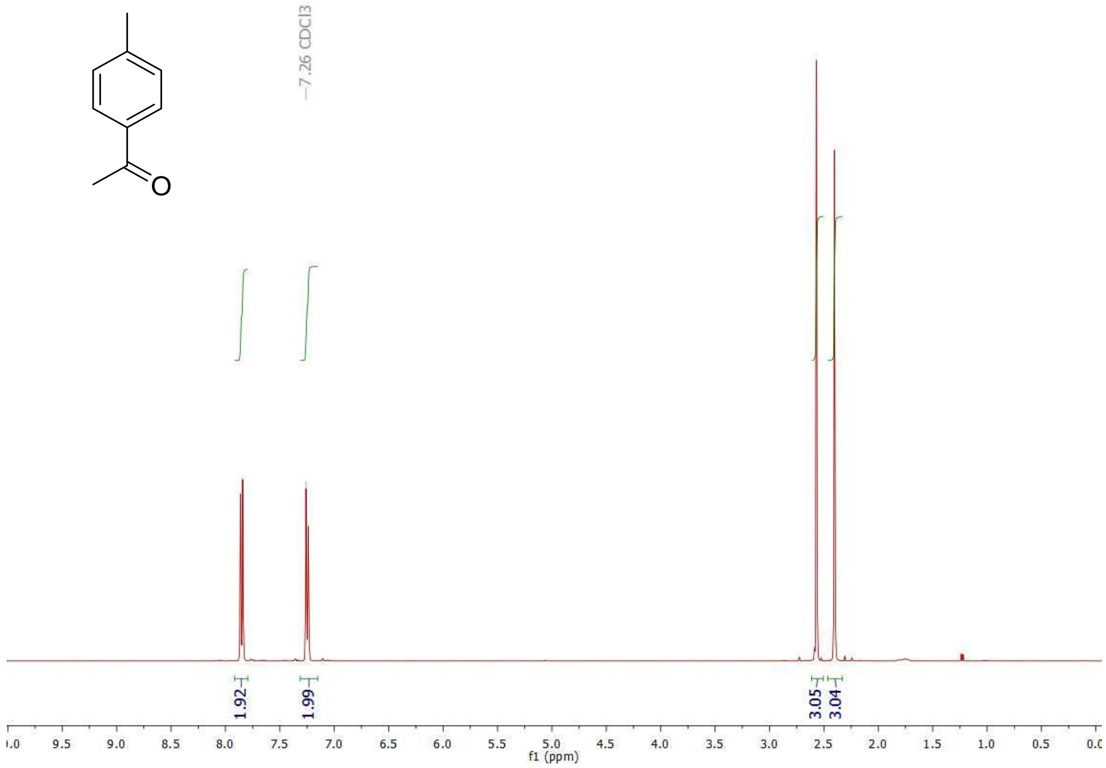

Figure S6. ^{13}C NMR spectrum of *p*-methylacetophenone.

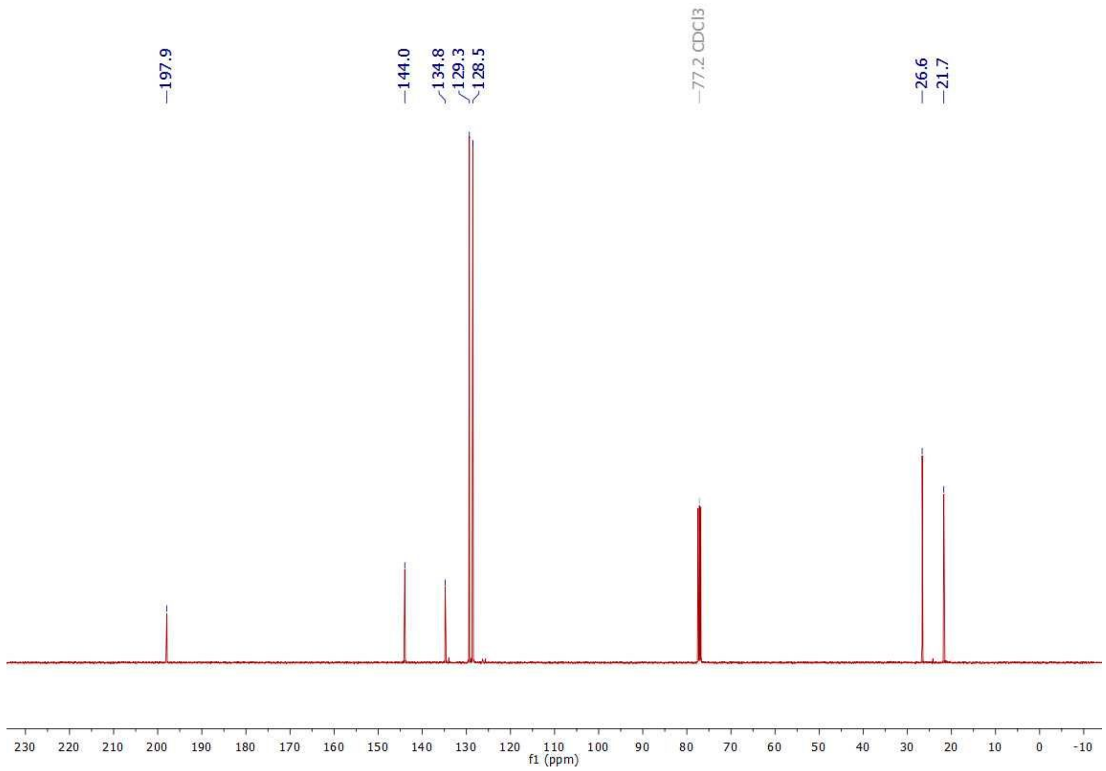

Figure S7. ^{1}H NMR spectrum of *p*-toluic acid.

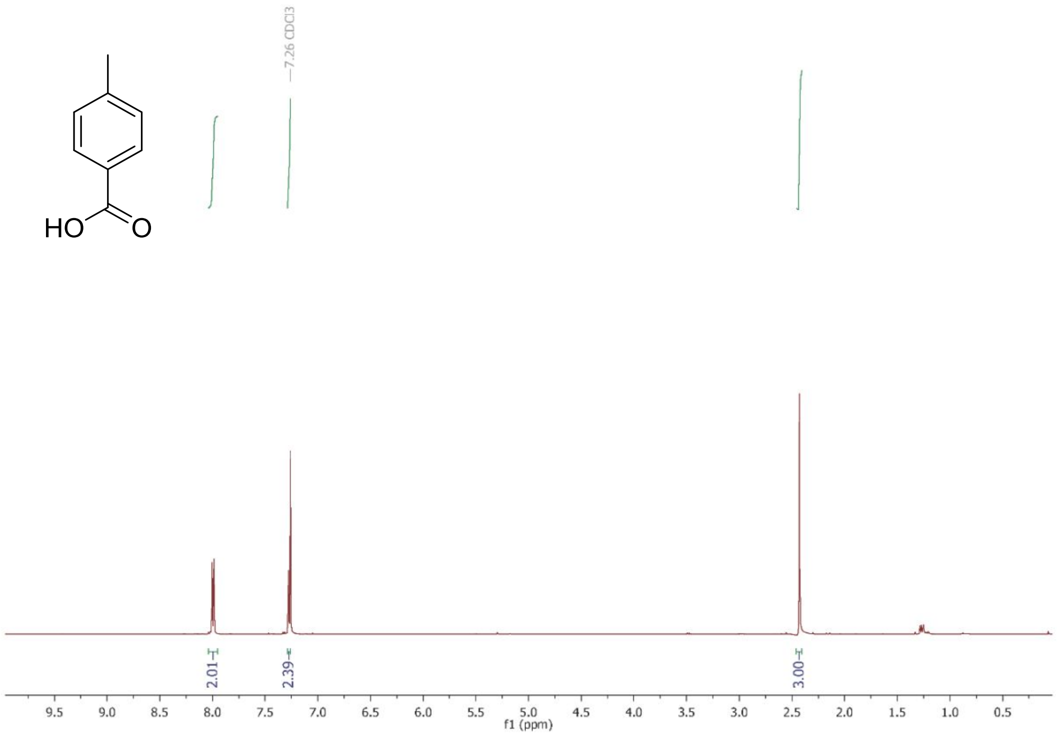

Figure S8. ^{13}C NMR spectrum of *p*-toluic acid.

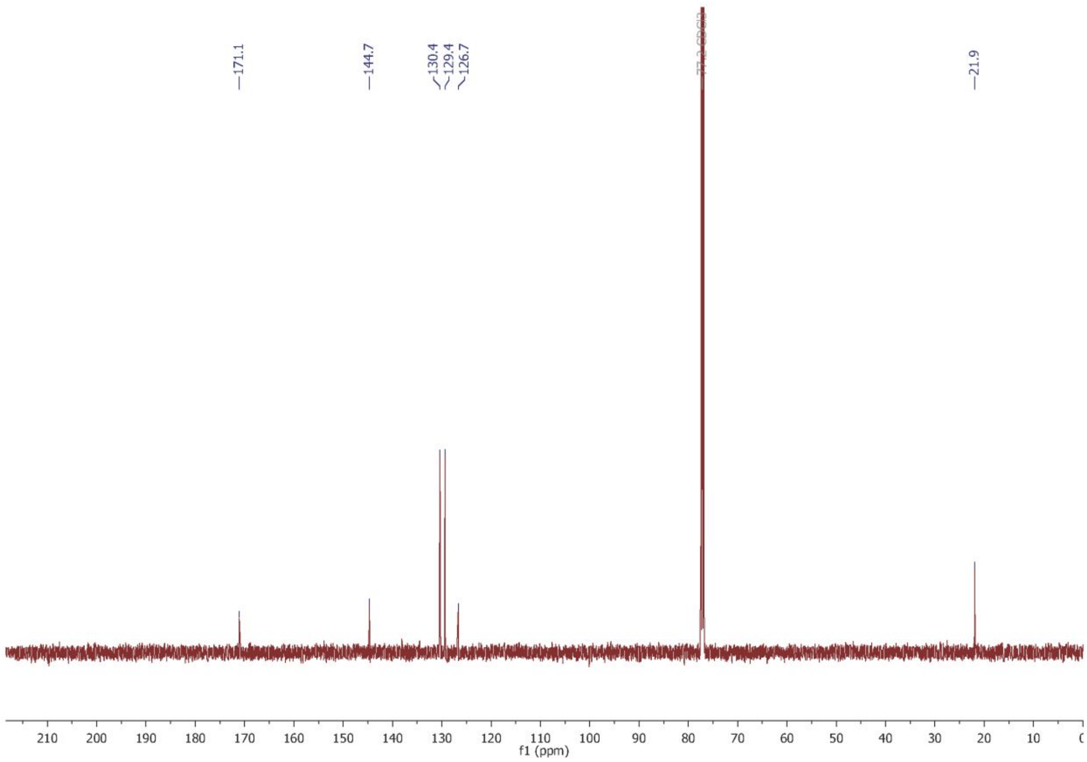

Figure S9. ^{1}H NMR spectrum of terephthalic acid.

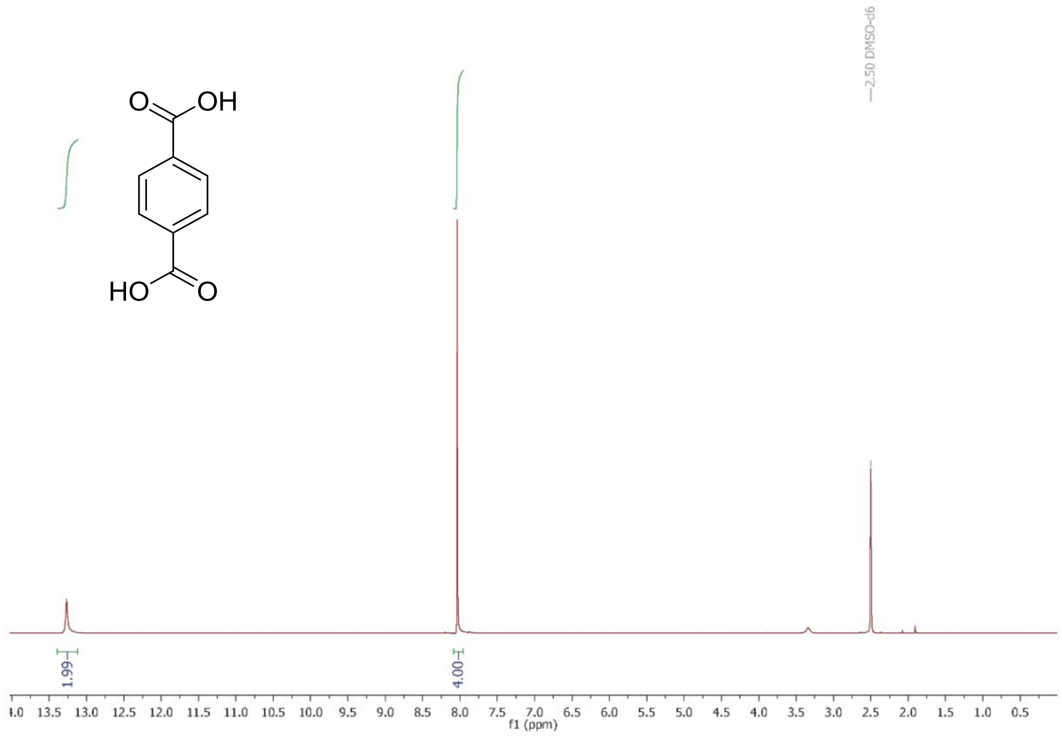

Figure S10. ^{13}C NMR spectrum of terephthalic acid.

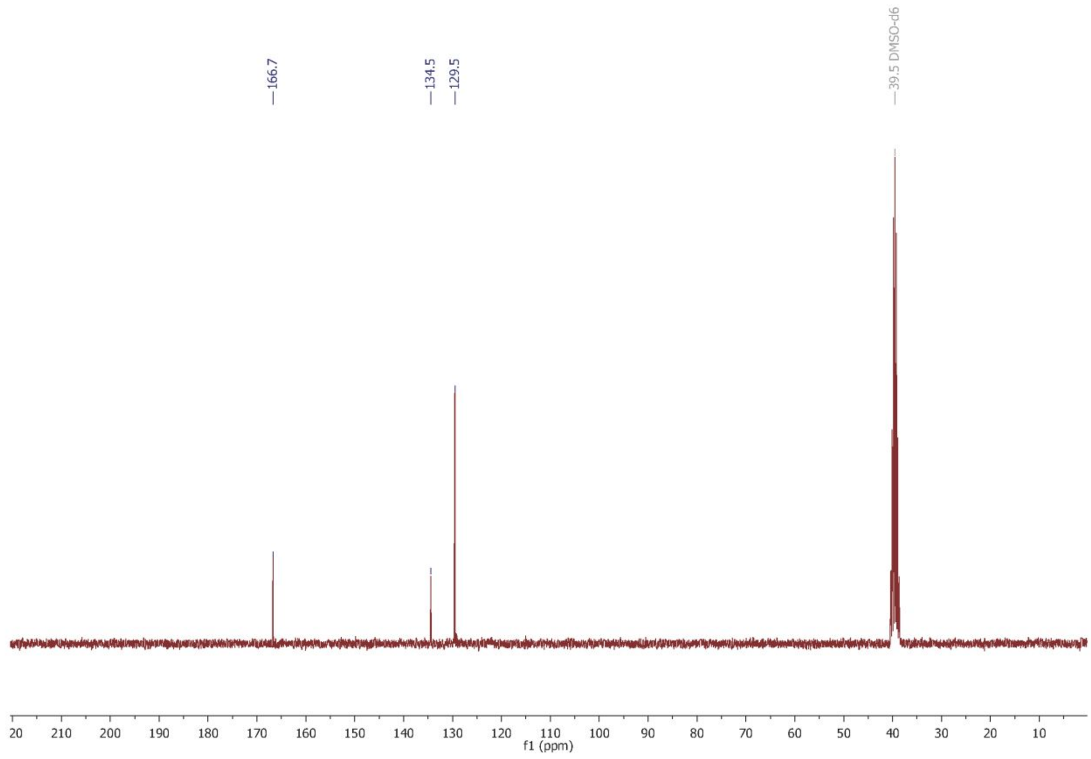

References

[1] A. Arneth, R. K. Monson, G. Schurgers, Ü Niinemets, P. I. Palmer, *Atmos. Chem. Phys.,* 2008, **8**, 4605–4620.
[2] E4tech, RE-CORD and WUR "From the Sugar Platform to biofuels and biochemicals", Final report for the European Commission, contract No. ENER/C2/423-2012/SI2.673791, 2015.
[3] K. K. Miller, P. Zhang, Y. Nishizawa-Brennen, J. W. Frost, *ACS Sustainable Chem. Eng.* 2014, **2**, 2053–2056.
[4] P. Zhang, V. Nguyen, J. W. Frost, *ACS Sustainable Chem. Eng.*, 2016, **4**, 5998−6001
[5] S. Bérard, C. Vallée, D. Delcroix, *Ind. Eng. Chem. Res.,* 2015, **54**, 7164−7168.
[6] T. Pfennig, R. L. Johnson, B. H. Shanks, *Green Chem*., 2017, **19**, 3263-3271.
[7] E4tech, "UK Top Bio-based Chemicals Opportunities", Report for LBNet, 2017
[8] D. I. Collias, A. M. Harris, V. Nagpal, I. W. Cottrell, M. W. Schultheis, *Ind. Biotechnol.*, 2014, **10**, 91–105.
[9] D. Kong, E. Tsang; I. Teixeria, Q. Song, DE102016219809A1, 2016.
[10] M. W. Peters, J. D. Taylor, M. Jenni, L. E. Manzer, D. E. Henton, US 2011/0087000 A1, 2011.
[11] J. W. Frost, A. Miermont, D. Schweitzer, V. Bui, D.A. Wicks, US 8,367,858/B2, 2013.
